# Impact of *Schistosoma mansoni* on Malaria Transmission in Sub-Saharan Africa

**DOI:** 10.1371/journal.pntd.0003234

**Published:** 2014-10-16

**Authors:** Martial L. Ndeffo Mbah, Laura Skrip, Scott Greenhalgh, Peter Hotez, Alison P. Galvani

**Affiliations:** 1 School of Public Health, Yale University, New Haven, Connecticut, United States of America; 2 National School of Tropical Medicine, and Sabin Vaccine Institute and Texas Children's Hospital Center for Vaccine Development, Baylor College of Medicine, Houston, Texas, United States of America; University of Washington, United States of America

## Abstract

**Background:**

Sub-Saharan Africa harbors the majority of the global burden of malaria and schistosomiasis infections. The co-endemicity of these two tropical diseases has prompted investigation into the mechanisms of coinfection, particularly the competing immunological responses associated with each disease. Epidemiological studies have shown that infection with *Schistosoma mansoni* is associated with a greater malaria incidence among school-age children.

**Methodology:**

We developed a co-epidemic model of malaria and *S. mansoni* transmission dynamics which takes into account key epidemiological interaction between the two diseases in terms of elevated malaria incidence among individuals with *S. mansoni* high egg output. The model was parameterized for *S. mansoni* high-risk endemic communities, using epidemiological and clinical data of the interaction between *S. mansoni* and malaria among children in sub-Saharan Africa. We evaluated the potential impact of the *S. mansoni*–malaria interaction and mass treatment of schistosomiasis on malaria prevalence in co-endemic communities.

**Principal Findings:**

Our results suggest that in the absence of mass drug administration of praziquantel, the interaction between *S. mansoni* and malaria may reduce the effectiveness of malaria treatment for curtailing malaria transmission, in *S. mansoni* high-risk endemic communities. However, when malaria treatment is used in combination with praziquantel, mass praziquantel administration may increase the effectiveness of malaria control intervention strategy for reducing malaria prevalence in malaria- *S. mansoni* co-endemic communities.

**Conclusions/Significance:**

Schistosomiasis treatment and control programmes in regions where *S. mansoni* and malaria are highly prevalent may have indirect benefits on reducing malaria transmission as a result of disease interactions. In particular, mass praziquantel administration may not only have the direct benefit of reducing schistosomiasis infection, it may also reduce malaria transmission and disease burden.

## Author Summary

Malaria and *Schistosoma mansoni* are co-endemic in many regions of sub-Saharan Africa. Evidence from clinical and epidemiological studies support the hypothesis that concurrent infection with *S. mansoni* is associated with greater malaria incidence among school-age children. We use mathematical modeling to evaluate the epidemiological impact of *S. mansoni* infection on malaria transmission in sub-Saharan Africa. Using epidemiological data on the increased risk of malaria incidence in *S. mansoni* endemic communities from Senegal, we developed a co-epidemic model of malaria and *S. mansoni* transmission dynamics to address key epidemiological interactions between the two diseases. Parameterizing our model for *S. mansoni* high-risk endemic communities, we show that the interaction between *S. mansoni* and malaria may reduce the effectiveness of malaria treatment for curtailing malaria transmission. Moreover, we show that in addition to reducing schistosomiasis health burden, mass praziquantel administration will generate indirect benefit in terms of reducing malaria transmission and disease burden in *S. mansoni*–malaria co-endemic communities. Our findings indicate the possible benefit of scaling up schistosomiasis control efforts in sub-Saharan Africa, and especially in areas were *S. mansoni* and malaria are highly prevalent.

## Introduction

Malaria is highly endemic throughout sub-Saharan Africa in which 85% of global malaria cases and 90% of malaria deaths occur [1]. *Schistosoma mansoni* (the causative agent of intestinal schistosomiasis) is likewise prevalent in many sub-Saharan African countries [2], [3], accounting for approximately one-third of the total cases of schistosomiasis in the region [4]. Both malaria and intestinal schistosomiasis share similar epidemiological distributions and present challenges to public health and socio-economic development throughout these regions [5]. Due to their coendemicity, there has been increased investigation into the interactive pathology between malaria and *S. mansoni* [6]–[9].

Heavy *S. mansoni* infections have been found to be associated with a significant increase in the incidence of malaria among school-age children [6]. While the mechanism responsible for the exacerbation of malaria in individuals infected with *S. mansoni* is not yet fully understood [7], [9], the interactions between the two diseases are possibly driven by countering effects the parasites have on immunological cytokines [10], [11]; that is, *S. mansoni* may alter the balance between Th1 and Th2 type immune responses [12]–[14] which reduces immunological control of malaria, although other mechanisms are possible.

Artemisinin-based combination therapies (ACT) are increasingly used as first-line treatment against malaria in sub-Saharan Africa [15], [16]. ACT is an efficacious drug regimen that reduces the risk of malaria-induced morbidity and mortality as well as malaria transmission from humans to vectors [17], [18]. For the control of schistosomiasis, current World Health Organization (WHO) guidelines recommend frequent mass administration of praziquantel, a highly effective and relatively inexpensive anti-schistosomal agent [19], to school-age children or to entire communities depending on schistosomiasis prevalence and available resources [20]. However, the adoption of mass praziquantel administration remains suboptimal in sub-Saharan Africa mainly due to limited drug availability, even as the schistosomiasis disease burden continues to rise [21]. Mass praziquantel treatment coverage and compliance may vary substantially from one schistosomiasis endemic area to another.

We evaluated how *S. mansoni* infection and mass praziquantel administration may affect the dynamics and control of malaria in co-endemic communities. In the absence of field studies that directly measure the effect of schistosomiasis control on malaria transmission and progression [6], [22]–[25], we address this question by using epidemiological and clinical findings that estimate the elevation in relative risk of malaria attributable to *S. mansoni* infection [6] to develop a mathematical model of the joint dynamics of malaria and *S. mansoni* among children. We use this model to evaluate the inter-dependent impact of *S. mansoni* on malaria infection and the potential impact of schistosomiasis and malaria treatment for reducing malaria transmission.

## Methods

To quantify the potential impact of *S. mansoni* infection and mass praziquantel administration on malaria transmission, we developed a compartmental deterministic model for co-endemic communities in sub-Saharan Africa. Specifically, our model describes the joint dynamics of malaria and *S. mansoni* transmission among children younger than 15 years old. This age group is the most at risk for both malaria and schistosomiasis transmission in sub-Saharan Africa [22].

### Model

We developed a mathematical model of the interplay between malaria and *S. mansoni*. Malaria transmission was modeled as follows [26]: At each point in time people can be in one of six infectious states – susceptible (*S*), treated symptomatic disease (*T*), untreated symptomatic disease (*D*), asymptomatic patent infection (*A*), asymptomatic sub-patent infection (*U*) and protected by a period of prophylaxis from treatment (*P*). We assumed that individuals entered the model susceptible and become infected at a rate determined by the force of infection in the population given by 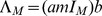, where 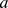 represents the biting rate on humans by a female mosquito, 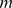 is the density of mosquitoes per human, 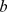 is the probability of successful human inoculation upon an infectious bite, and 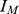 the proportion of infectious mosquitoes in the vector population. Upon infection, individuals either develop symptomatic disease (with a probability *Φ*) or develop patent asymptomatic infection (*1−Φ*). Those who develop symptomatic disease have a fixed probability (*f_T_*) of being treated successfully with an ACT (*T*), in which case they clear infection at a rate *r_T_* and enter a period of prophylactic protection (*P*) before returning (*r_P_*) to being susceptible to new infection. Those who fail treatment (*1−f_T_*) are assumed to eventually clear disease (*D*) and become patently asymptomatic (*A*) at rate *r_D_*. From patent asymptomatic infection, individuals will move to a sub-patent stage (*U*) at a rate *r_A_* and then clear infection at rate *r_U_* and individuals return to being susceptible. The force of infection of malaria on the mosquito population, 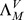, was given by the product of host biting rate per mosquito, probability of mosquito infection upon biting an infectious human 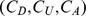, and the proportion of infected individuals at each infectious stage (*D, A, U*). The intensity of malaria transmission is represented as the annual entomological inoculation rate (AEIR), defined as the product of the human biting rate of mosquitoes and the proportion of mosquitoes that are infectious. AEIR is measured in the number of infective bites per person per year (ibpy). Here malaria prevalence refers to any level of parasitaemia rather than symptomatic disease alone.

For *S. mansoni* transmission, we assumed that at each point in time people can be in one of three states – susceptible (*S*), infected with low egg output (*I_L_*), and infected with high egg output (*I_H_*) [27]. Likelihood of schistosomiasis transmission from humans to snails depends on worm burden and mean egg production per worm. For the sake of simplicity, egg production was not explicitly modeled. However consistent with previous schistosomiasis modeling studies, we used transmission rates that implicitly account for egg production rate per worm, contact with infested waters, and probability of worm establishment per contact [27], [28]. We assumed that individuals entered the model susceptible and become infected with an initially low egg output at a transmission rate 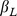. Individuals with low egg output may then transition to high egg output at a transmission rate 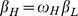, where 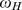 determines the rate of transition to a high egg output from a low egg output relative to 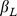. We assumed the individuals infected with low egg output infect susceptible snails at a transmission rate 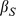, and individuals with high egg output infect snails at a transmission rate 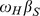, where 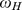 is the relative increase of transmission rate to snails for high egg output individuals relative to low egg output individuals. Because rates of schistosomiasis reinfection are very high in endemic areas, we assumed that there is no natural recovery for *S. mansoni* infected individuals, and that without treatment infected individuals with a high egg output will not transition to a low egg output. We incorporated annual praziquantel treatment into the model by assuming that treatment has an efficacy of 70% [29], [30]. We assumed that upon treatment, 70% of individuals with low egg output will recover from infection, and 70% of individuals with high egg output will either recover from infection or have their egg output reduced to a low level, such that 40% of treated high egg output will recover from infection, while 30% will have their egg output reduced to a low level [29], [30]. We evaluated the potential impact of deworming through mass drug administration with praziquantel on malaria prevalence by considering different levels of treatment coverage ranging from 30–80%.

Individuals can be infected with malaria only, *S. mansoni* only, or dually infected with malaria and *S. mansoni*. The model captures the epidemiological interaction between the two diseases in terms of *S. mansoni* enhancing susceptibility to malaria denoted as 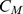 and parameterized from epidemiological field data [6] (Table 1). We focused on communities in which malaria and *S. mansoni* are co-endemic, and considered variation in malaria transmission intensity by varying the AEIR from 1 ibpy to 500 ibpy [31]. We present results obtained at endemic equilibrium. A detailed description of the model is given in the Supplement Material and an overview of parameters and values used to generate the model outcomes are given in Table 1.

**Table 1:** Parameter definitions of our *Schistosoma mansoni*-malaria co-infection model.

Parameter	Definition	Value (95%CI)	Ref
*μ_d_*	Natural death rate of host	0.05 yr^−1^	—
*a*	Biting rate on humans by a female mosquito	0.67 Day^−1^	[71]
*b*	Probability of successful human inoculation upon an infectious bite	0.25	[38]
*m*	Density of mosquitoes per human	varied†	—
*φ*	Probability of becoming symptomatic case upon infection (susceptibility)	0.72	[72]
*r_D_* ^−1^	Duration of symptomatic malaria	5 Days	[73]
*r_A_* ^−1^	Duration of asymptomatic malaria	180 Days	[26]
*r_U_* ^−1^	Duration of sub-patent malaria	180 Days	[74]
*r_T_* ^−1^	Duration of clinical malaria upon chemotherapy	5 Days	[75]
*r_P_* ^−1^	Duration of prophylaxis from malaria treatment	20 Days	[75], [76]
*f_T_*	Proportion of symptomatic malaria cases treated effectively	0.5 (0–0.9)	[77], [78]
*μ_M_*	Mosquito natural mortality rate	1/8 Day^−1^	[26]
*t_incub_*	Mosquito incubation period	10 Days	[26]
*C_D_*	Probability of mosquito infection upon biting a human in state untreated clinical disease	0.3	[79]
*C_A_*	Probability of mosquito infection upon biting a human in state Asymptomatic patent infection	0.1	[79]
*C_U_*	Probability of mosquito infection upon biting a human in state sub-patent infection	0.05	[79]
*C_M_*	Enhancement of malaria susceptibility due to high worm burden	1.85 (1.16–2.74)	[6]
*β_S_*	Human low worm burden to snails transmission	0.02 (0.01–1.88) yr^−1^	estimated*
*β_L_*	Snail to human transmission from no infection to low worm burden	7.08 (0.2–9.84) yr^−1^	estimated*
*ε_H_*	Human high worm burden to snails transmission relative to *β_S_*	1.91 (1.0–18.3)	estimated*
*ω_H_*	Snail to human transmission from low to high worm burden relative to *β_L_*	0.77 (0.04–0.97)	estimated*
*δ_S_*	Snail natural mortality rate	0.17 yr^−1^	[80]
*C_T_*	Mass praziquantel treatment coverage	30–80%	—

*Parameters were estimated suing a Bayesian Melding procedure [81], [82] to fit the *S. mansoni* dynamic model to prevalence data for high-risk endemic communities. In high-risk communities, the overall *S. mansoni* prevalence was varied from 40–80% and the high worm burden prevalence was varied from 15–60% [6], [17], [83], [84]. High worm burden was defined as having a *S. mansoni* load exceeding 1000 eggs/g of stool [6].

† Density of mosquitoes per human was varied so as to account for different value of the annual entomological inoculation rate.

## Results

**Figure 1:**
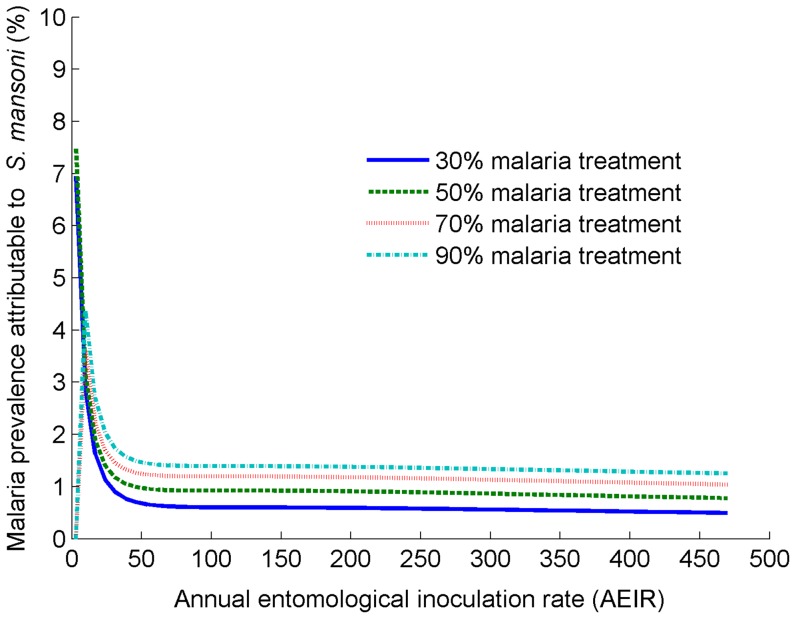
Malaria prevalence attributable to *S. mansoni* in a range of malaria transmission settings. The prevalence attributable to *S. mansoni* is the difference between the equilibrium malaria prevalence in the presence of interaction and that in the absence of interaction between *S. mansoni* and malaria. *S. mansoni* high worm burden was assumed to increase the risk of malaria infection by 85% 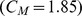, consistent with epidemiological studies [6].

By comparing malaria prevalence in the presence and absence of *S. mansoni* co-endemicity, we showed that the impact of schistosomiasis co-infection on increasing malaria prevalence was higher in areas of low malaria transmission than areas of high malaria transmission (Figures 1 & S1). For example, disease interaction was shown to increase malaria prevalence by 3.0–4.5% for an AEIR of 10 ibpy and by 0.6–1.5% for an AEIR of 100 ibpy, depending on malaria treatment coverage, ranging from 30–90% (Figure 1). The effect of *S. mansoni* co-infection on malaria prevalence plateaued from 100 ibpy upwards (Figure 1).

**Figure 2:**
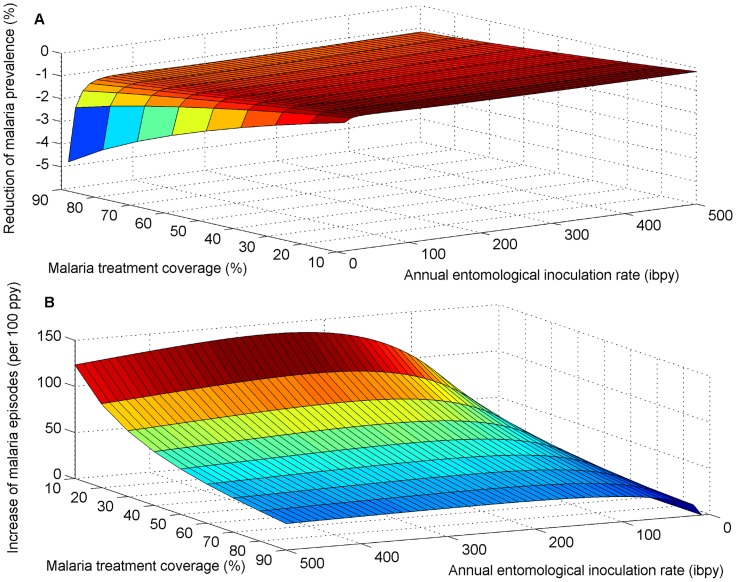
The impact of the interaction between *S. mansoni* and malaria on the effectiveness of ACT for reducing malaria prevalence and symptomatic malaria episodes. We compared (A) the proportional reduction of malaria prevalence with and without interaction and (B) the increase in symptomatic episodes of malaria due to elevated susceptibility to malaria mediated by *S. mansoni* infection. *S. mansoni* high worm burden was assumed to increase the risk of malaria infection by 85% 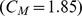, consistent with epidemiological studies [6].

We also found that the interaction between malaria and *S. mansoni* may reduce the effectiveness of malaria treatment for decreasing malaria prevalence (Figure 2). For an AEIR of 100 ibpy, *S. mansoni* co-infection was shown to decrease the proportional reduction of malaria prevalence due to treatment by 1.3% for 90% treatment coverage, 1% for 60% treatment coverage, and 0.5% for 30% treatment coverage (Figure 2A). For 90% malaria treatment coverage, *S. mansoni* co-infection increases symptomatic malaria episodes by 29 episodes per 100 people annually, by 45 episodes per 100 people annually for 60% treatment coverage, and 93 episodes per 100 people annually for 30% treatment coverage (Figure 2B).

For an AEIR of 10 ibpy, disease interaction was shown to decrease the proportional reduction of malaria prevalence due to treatment by 2.5% for 90% treatment coverage, 1.4% for 60% treatment coverage, and by 0.6% for 30% treatment coverage (Figure 2A). For 90% malaria treatment coverage *S. mansoni* co-infection increases symptomatic malaria episodes by 11 episodes per 100 people annually, by 16 episodes per 100 people annually for 60% treatment coverage, and 21 episodes per 100 people annually for 30% treatment coverage (Figure 2B).

**Figure 3:**
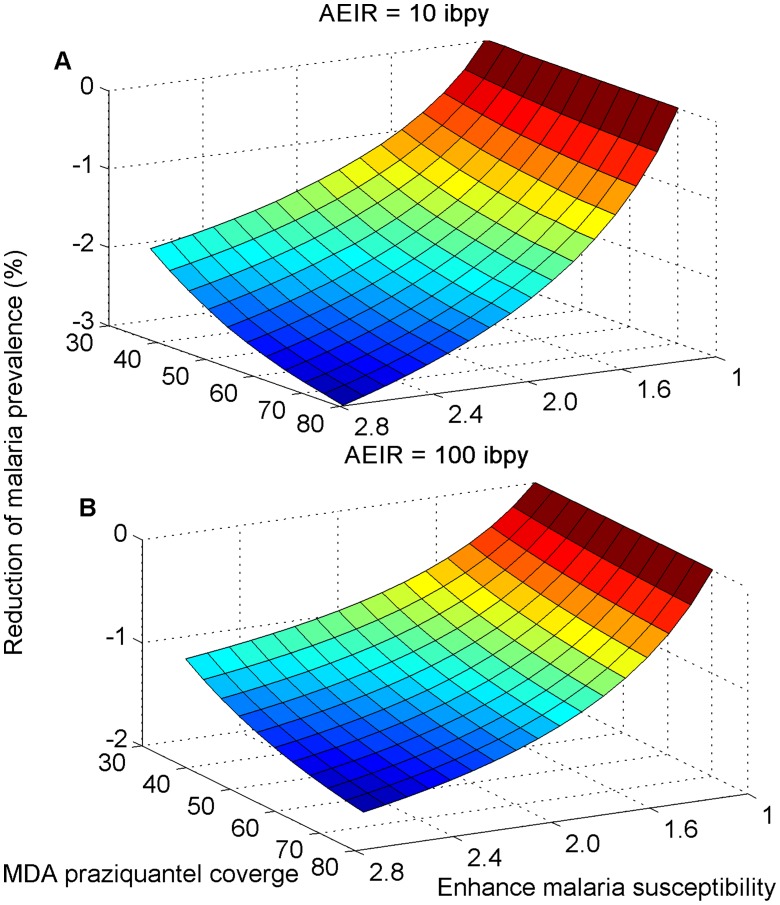
The impact of mass praziquantel administration on malaria prevalence over a six-year intervention period. The proportion of symptomatic malaria cases that received treatment was 70%. Interaction between *S. mansoni* and malaria and the effect on malaria prevalence for annual entomological inoculation rate (AEIR) equals (A) 10 infective bites per person annually and (B) 100 infective bites per person annually.

When ACT is used in combination with annual mass praziquantel administration, we showed that the intervention was more effective in reducing malaria prevalence and that this effectiveness increases both with the coverage of praziquantel and with the increased susceptibility to malaria infection due to *S. mansoni* (Figure 3). This increase in effectiveness was more pronounced in areas of low malaria transmission intensity (Figure 3A) than in areas of high transmission intensity (Figure 3B). The interaction between *S. mansoni* and malaria generated an additional indirect benefit for mass praziquantel administration by reducing malaria prevalence (Figure 3).

## Discussion

We developed a co-epidemic model of malaria and *S. mansoni* transmission dynamics to take into account elevated susceptibility to malaria mediated by *S. mansoni* infection. We used this model to investigate the potential effect of malaria-*S. mansoni* interaction on the effectiveness of ACT and mass praziquantel administration for schistosomiasis for reducing malaria prevalence in co-endemic communities. Our results suggested that co-infection with schistosomiasis in low malaria transmission settings increases malaria prevalence. We further showed that in the absence of mass praziquantel administration, the interaction between *S. mansoni* and malaria may have contributed to reductions in population-level effectiveness of malaria treatment in areas of stable malaria transmission. In regions of low malaria treatment coverage, co-infection with schistosomiasis led to the greatest increase in per person malaria episodes, independent of whether malaria transmission was high or low. Our finding is consistent with epidemiological observations and laboratory studies that have suggested that presence of *S. mansoni* infections may affect the efficacy of malaria control measures, including a potential vaccine in co-endemic communities [9], [32]. The interaction between the two diseases may increase the health benefits of mass praziquantel administration by generating the additional indirect benefit of reducing malaria transmission in co-endemic communities. Our results indicated that this benefit was particularly strong in low malaria transmission regions that experienced increased malaria susceptibility due to schistosomiasis co-infection. Malaria is associated with a Th1 immune response [12], while *S. mansoni* infection is associated with a Th2 response and had been demonstrated to impair immune responses to malaria [11], [13]. By reducing *S. mansoni* worm burden of infected individuals, praziquantel treatment may reduce the Th2 immune response associated with *S. mansoni* infection which may in turn result in a shift in the Th1/Th2 immune balance [14], [33] towards the Th1 response that protects against malaria parasite. Though our study focused on *Plasmodium falciparum*, our results may be applicable to other forms of malaria such as *Plasmodium ovale* and *Plasmodium vivax*, which may also interact with *S. mansoni*. Prototype vaccines for both malaria [34] and *S. mansoni* intestinal schistosomiasis [35] are under development, such that the two vaccines could be co-formulated or combined [36]. Our results suggest that a co-formulated or combined vaccine may be more efficacious in reducing malaria transmission in *S. mansoni* endemic communities than a vaccine targeting malaria alone.

In addition to increasing malaria incidence, clinical studies have shown that malaria–*S. mansoni* co-infection may exacerbate clinical manifestations of both diseases [14], [33], [37]. These additional impacts were not factored into our model, making our predictions of the effectiveness of joint programs of ACT and praziquantel conservative. Our model also did not account for malaria age-dependent immunity [26], [38]. Age-dependent malaria immunity is less important among children than adults, however, and it is even less relevant in areas of low malaria transmission [26], [38], [39]. We anticipate that accounting for age-dependent malaria immunity would only have a marginal quantitative effect on our results, such that the findings would remain qualitatively unchanged. Malaria and *S. mansoni* may differ in their distribution of disease intensity, prevalence, and morbidity, with some portion of the population being at higher risk than others [33]. Therefore, the magnitude of the interaction between malaria and *S. mansoni* on malaria transmission dynamics may vary from one risk group to another. Given that data on risk group specific interaction between malaria and *S. mansoni* are not available, our model only accounted for elevated malaria susceptibility from *S. mansoni* high egg output. Future studies could account for heterogeneity in malaria intensity and prevalence.

Currently, there is debate surrounding the extent and direction of the effects of malaria and co-infection with different helminth species on human hosts [7], [33], [40]. Apparent contradictions arising from clinical and epidemiological studies may be resolved by the possibility of species-specific effects of helminth infections on malaria [7], [40], [41]. As well as qualitatively different interactions for different worm burdens For example, *Ascaris* has been associated with protection from severe malaria complications [7]. Conversely, epidemiological studies have suggested that hookworm elevates malaria prevalence [42] and exacerbates malaria-induced anaemia [22], [43]. Similarly, *S. mansoni* has been shown to be associated with increased malaria incidence [6] and exacerbation of hepatosplenomegaly [37], [44] and anemia [45] in individuals co-infected with malaria. It has also been reported that children with low (but not high) *S. haematobium* infection intensity co-infected with malaria have significantly lower *P. falciparum* parasitemia than worm-free individuals [46]. This observation implies that the interaction between *P. falciparum* and *S. haematobium* may have contributed to lower malaria prevalence in *S. haematobium* low risk endemic communities, but that the reverse could be the case in *S. haematobium* high risk communities. Additionally, malaria-schistosomiasis coinfection may have opposite effect on malaria transmission in *S. haematobium* compare to *S. mansoni* endemic communities. Therefore, in *S. mansoni* - *S. haematobium* co-endemic communities [47], [48], schistosomiasis control may have a very complex impact on malaria transmission. Further studies are needed on the interaction of *S. mansoni* and *S. haematobium* and their potential impact on malaria transmission. Future transmission models on this topic could also account for worm mating probability and density dependent effects on egg output per worm, which can affect schistosomiasis transmission [49], [50].

Polyparasite helminth infections and malaria co-infection are widespread throughout sub-Saharan Africa [22], [51], [52]. Therefore, studies investigating how co-infection affects the course of each infection, as well as immune responses, are fundamental to understand the potential additional benefits or perverse effects of mass drug administration and control programmes for tropical diseases. There are myriad examples of parasitic co-endemicity and co-infections affecting health outcomes in sub-Saharan Africa. For example malaria and hookworm co-infections [22], [53] as well as and *S. mansoni* and hookworm co-infections [54] can lead to severe anemia. A new modeling study on the interaction between lymphatic filariasis and malaria that takes into account increase in vector mortality due to lymphatic filariasis prevalence in mosquito and antagonistic Th1/Th2 immune response in co-infected host has shown that control strategies that reduce lymphatic filariasis transmission could potentially increase malaria prevalence [55]. Similarly, some studies have indicated that antimalarial bednets may reduce transmission from lymphatic filariasis transmitted by anopheles mosquitoes [56], [57]. In addition, *S. haematobium* is interacting with HIV by increasing susceptibility to HIV infection through lesions and inflammation of genital track and immunomodulation effects [58]. Two large studies in Zimbabwe and Tanzania found that women with genital schistosomiasis have a 3–4 fold increased odds of having HIV compared to women without genital schistosomiasis [59], [60]. Subsequent models have shown that female genital schistosomiasis (caused by *S. haematobium*) control strategies could reduce HIV transmission [61], [62], in co-endemic communities. One of the limitations of our study was that we did not examine the relationship between *S. haematobium* and malaria. Future studies could investigate the interaction between malaria and *S. haematobium*, as well as other helminths including hookworm. Such studies could also investigate low risk schistosomiasis communities where, because of the low rate of schistosomiasis reinfection, the sequential order of infection between malaria and schistosomiasis may impact the co-infections of schistosomiasis on malaria transmission.

Clinical studies have shown that ACT used in combination with praziquantel may reduce both the malaria and the schistosomiasis health burden in co-infected individuals [63]–[66], and that artemisinin-based therapy may have indirect benefits for reducing schistosomiasis health burden [63].

Additional drug interaction studies may be required if ACT and praziquantel are combined for purposes of mass drug administration. In an experimental rat model of clonorchiasis, combinations of praziquantel and artemisinins produced both synergistic and antagonistic effects depending on the doses administered [67]. In humans infected with *S. japonicum* in China, it was noted that the combination of artemether and praziquantel chemotherapy did not improve treatment efficacy relative to praziquantel alone [68], while in Africa (Cote d'Ivoire) the addition of mefloquine-artesunate did not increase the efficacy of praziquantel against *S. haematobium* infection [69].

Integrating mass screening and treatment for malaria using ACT with mass drug administration of praziquantel could contribute to reducing both malaria and schistosomiaisis transmission in sub-Saharan Africa. Therefore, future studies would investigate the complementary effects of ACTs and mass praziquantel administration for reducing both malaria and schistosomiasis transmission in co-endemic communities. Immunological studies have suggested that praziquantel treatment in malaria-schistosomiasis co-endemic communities may alter the immune response of treated individuals, making them less susceptible to malaria infection [70]. However, more studies are needed to confirm this impact of praziquantel treatment.

Our results suggest that in *S. mansoni* endemic areas, mass treatment of schistosomiasis may not only have a direct benefit of reducing schistosomiasis infection, it may also reduce malaria prevalence and disease burden. This reduction of malaria prevalence was higher in areas of low malaria transmission intensity, but less pronounced in areas of high transmission intensity (AEIR greater than 100 ibpy). Additional epidemiological and clinical data on malaria–*S. mansoni* co-infection to determine influence on immune responses and duration of malaria infection are needed to fully evaluate the potential effects of *S. mansoni* and schistosomiasis control strategies on malaria.

## Supporting Information

Figure S1Difference in malaria prevalence attributable to *S. mansoni* at different level of malaria treatment coverage for a wide range of malaria and *Schistosoma mansoni* transmission settings. Malaria transmission settings were obtained by varying the AEIR, whereas *S. mansoni* transmission settings were obtained by sampling schistosomiasis transmission parameters over the ranges of values given in Table 1. *S. mansoni* high worm burden is assumed to increase the risk of malaria infection by 85% 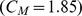. Interaction between malaria and *S. mansoni* and the effect on (A) the difference in malaria prevalence attributable to *S. mansoni* for 70% malaria treatment coverage versus 50% treatment coverage, and (B) the difference between 90% malaria treatment coverage versus 70% coverage.(TIF)

Text S1 Supplementary Material for Epidemiological impact of *Schistosoma mansoni* on Malaria transmission in sub-Saharan Africa.
(DOC)
