# Supplementary figures and images for: Impact of Schistosoma mansoni on Malaria Transmission in Sub-Saharan Africa
Source: PLoS Negl Trop Dis. 2014 Oct 16;8(10):e3234. doi: 10.1371/journal.pntd.0003234 (PMC4199517; doi:10.1371/journal.pntd.0003234)

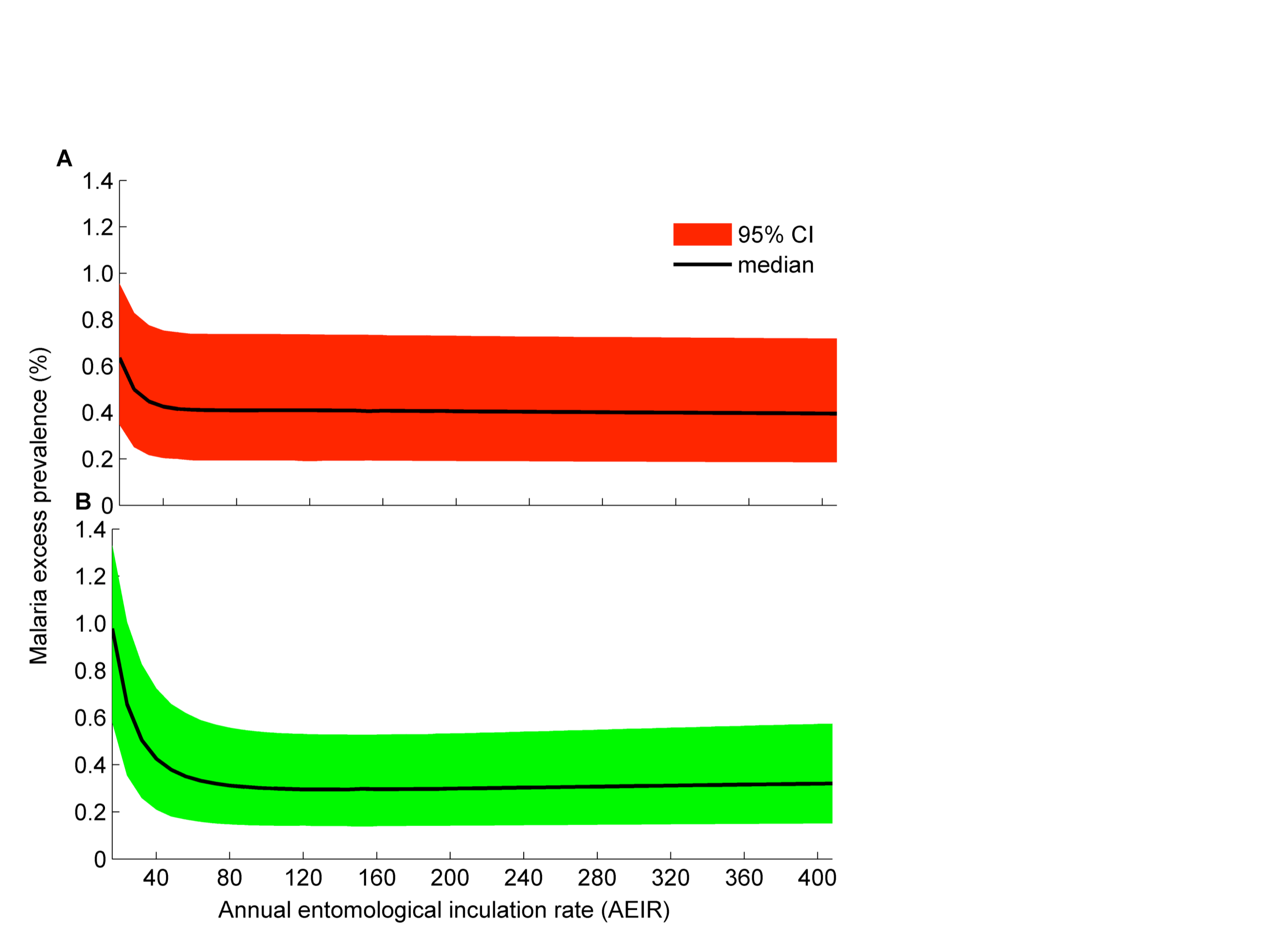

Supplement: Figure S1 — Difference in malaria prevalence attributable to S. mansoni at different level of malaria treatment coverage for a wide range of malaria and Schistosoma mansoni transmission settings. Malaria transmission settings were obtained by varying the AEIR, whereas S. mansoni transmission settings were obtained by sampling schistosomiasis transmission parameters over the ranges of values given in Table 1. S. mansoni high worm burden is assumed to increase the risk of malaria infection by 85% . Interaction between malaria and S. mansoni and the effect on (A) the difference in malaria prevalence attributable to S. mansoni for 70% malaria treatment coverage versus 50% treatment coverage, and (B) the difference between 90% malaria treatment coverage versus 70% coverage. (TIF) [file pntd.0003234.s001.tif]
