# Supplementary material for: Impact of Schistosoma mansoni on Malaria Transmission in Sub-Saharan Africa
Source: PLoS Negl Trop Dis. 2014 Oct 16;8(10):e3234. doi: 10.1371/journal.pntd.0003234 (PMC4199517; doi:10.1371/journal.pntd.0003234)
Supplement: Text S1 — Supplementary Material for Epidemiological impact of Schistosoma mansoni on Malaria transmission in sub-Saharan Africa. (DOC) [file pntd.0003234.s002.doc]

**Supplementary Material for**

**Epidemiological impact of *Schistosoma mansoni* on Malaria transmission in sub-Saharan Africa**

Martial L. Ndeffo Mbah, Laura Skrip, Scott Greenhalgh, Peter J. Hotez, Alison P. Galvani

**Schistosomiasis-malaria interaction model:**

We developed a mathematical model of the interplay between malaria and *Schistosoma mansoni*. Malaria transmission was modeled as follows [1]: People can be in one of six states – susceptible (S), treated symptomatic disease (T), untreated symptomatic disease (D), asymptomatic patent infection (A), sub-patent infection (U) and protected by a period of prophylaxis from treatment (P). For *S. mansoni* transmission, people can be in one of three states – susceptible (S), infected with low worm burden (I_L_), and infected with high worm burden (I_H_) [2]. Individuals can be infected with malaria only, *S. mansoni* only, or dually infected with malaria and *S. mansoni*. The co-infection model consists of twenty-two coupled nonlinear ordinary differential equations, eighteen of which describe the human population, two of which describe the malaria vector population (female *Anopheles* mosquitoes), and two of which describe the snail vector population for *S. mansoni*. The model calculates the extent of the epidemiological interaction between the two diseases based on the assumptions that high *S. mansoni* worm burden enhances susceptibility to malaria [3], and symptomatic malaria decreases an individual’s contact with infected waters.

$\frac{dS_{S}}{dt}=\delta_{S}(S_{S}+I_{S})-\lambda_{S}^{V}S_{S}-\delta_{S}S_{S}$ (1.1)

$\frac{dI_{S}}{dt}=\lambda_{S}^{V}S_{S}-\delta_{S}I_{S}$ (1.2)

$\frac{dS_{M}}{dt}=\mu_{M}(S_{M}+I_{M})-\Lambda_{M}^{V}S_{M}-\mu_{M}S_{M}$ (1.3)

$\frac{dI_{M}}{dt}=\Lambda_{M}^{V}S_{M}\psi-\mu_{M}I_{M}$ (1.4)

$\frac{dS}{dt}=\mu_{Pop}-\Lambda_{M}S-\mu_{d}S-\beta_{L}I_{S}S+T_{e}C_{T}I_{L}^{S}+\gamma_{H}C_{T}I_{H}^{S}+r_{U}U+r_{P}P$ (1.5)

$\frac{dI_{L}^{S}}{dt}=-\Lambda_{M}I_{L}^{S}-\mu_{d}I_{L}^{S}+\beta_{L}I_{S}(S-\omega_{H}I_{L}^{S})-T_{e}C_{T}I_{L}^{S}+r_{U}U_{L}^{S}+r_{P}P_{L}^{S}+\gamma_{L}C_{T}I_{H}^{S}$ (1.6)

$\frac{dI_{H}^{S}}{dt}=-C_{M}\Lambda_{M}I_{H}^{S}-\mu_{d}I_{H}^{S}+\beta_{L}I_{S}(\omega_{H}I_{L}^{S})-(\gamma_{H}+\gamma_{L})C_{T}I_{H}^{S}+r_{U}U_{H}^{S}+r_{P}P_{H}^{S}$ (1.7)

$\frac{dA}{dt}=(1-\varphi)\Lambda_{M}(S+U)-\varphi\Lambda_{M}A-r_{A}A+r_{D}D-\mu_{d}A-\beta_{L}I_{S}A+T_{e}C_{T}A_{L}^{S}++\gamma_{H}C_{T}A_{H}^{S}$ (1.8)

$\frac{dD}{dt}=\varphi(1-f_{T})\Lambda_{M}(S+U+A)-r_{D}D-\mu_{d}D-\beta_{L}I_{S}D+T_{e}C_{T}D_{L}^{S}+\gamma_{H}C_{T}D_{H}^{S}$ (1.9)

$\frac{dT}{dt}=\varphi(f_{T})\Lambda_{M}(S+U+A)-r_{T}T-\mu_{d}T-\beta_{L}I_{S}T+T_{e}C_{T}T_{L}^{S}+\gamma_{H}C_{T}T_{H}^{S}$ (1.10)

$\frac{dU}{dt}=r{}_{A}A-r_{U}U-\Lambda_{M}U-\mu_{d}U-\beta_{L}I_{S}U+T_{e}C_{T}U_{L}^{S}+\gamma_{H}C_{T}U_{H}^{S}$ (1.11)

$\frac{dP}{dt}=r{}_{T}T-r_{P}P-\mu_{d}P-\beta_{L}I_{S}P+T_{e}C_{T}P_{L}^{S}+\gamma_{H}C_{T}P_{H}^{S}$ (1.12)

$\frac{dA_{L}^{S}}{dt}=(1-\varphi)\Lambda_{M}(I_{L}^{S}+U_{L}^{S})-(\varphi\Lambda_{M}+r_{A}+\mu_{d}+T_{e}C_{T})A_{L}^{S}+r_{D}D_{L}^{S}-\beta_{L}I_{S}(A-\omega_{H}A_{L}^{S})+\gamma_{L}C_{T}A_{H}^{S}$(1.13)

$\frac{dA_{H}^{S}}{dt}=C_{M}(1-\varphi)\Lambda_{M}(I_{H}^{S}+U_{H}^{S})-(C_{M}\varphi\Lambda_{M}+r_{A}+\mu_{d}+(\gamma_{H}+\gamma_{L})C_{T})A_{H}^{S}+r_{D}D_{H}^{S}-\beta_{L}I_{S}(\omega_{H}A_{L}^{S})$(1.14)

$\frac{dD_{L}^{S}}{dt}=\varphi(1-f_{T})\Lambda_{M}(I_{L}^{S}+U_{L}^{S}+A_{L}^{S})-(r_{D}+\mu_{d}+T_{e}C_{T})D_{L}^{S}+\beta_{L}I_{S}(D-\omega_{H}D_{L}^{S})+\gamma_{L}C_{T}D_{H}^{S}$ (1.15)

$\frac{dD_{H}^{S}}{dt}=C_{M}\varphi(1-f_{T})\Lambda_{M}(I_{H}^{S}+U_{H}^{S}+A_{H}^{S})-r_{D}D_{H}^{S}-\mu_{d}D_{H}^{S}+\beta_{L}I_{S}(\omega_{H}D_{L}^{S})-(\gamma_{H}+\gamma_{L})C_{T}D_{H}^{S}$ (1.16)

$\frac{dT_{L}^{S}}{dt}=\varphi(f_{T})\Lambda_{M}(I_{L}^{S}+U_{L}^{S}+A_{L}^{S})-r_{T}T_{L}^{S}-\mu_{d}T_{L}^{S}+\beta_{L}I_{S}(T-\omega_{H}T_{L}^{S})-T_{e}C_{T}T_{L}^{S}+\gamma_{L}C_{T}T_{H}^{S}$ (1.17)

$\frac{dT_{H}^{S}}{dt}=C_{M}\varphi(f_{T})\Lambda_{M}(I_{H}^{S}+U_{H}^{S}+A_{H}^{S})-r_{T}T_{H}^{S}-\mu_{d}T_{H}^{S}+\beta_{L}I_{S}(\omega_{H}T_{L}^{S})-(\gamma_{H}+\gamma_{L})C_{T}T_{H}^{S}$ (1.18)

$\frac{dU_{L}^{S}}{dt}=r{}_{A}A_{L}^{S}-r_{U}U_{L}^{S}-\Lambda_{M}U_{L}^{S}-\mu_{d}U_{L}^{S}+\beta_{L}I_{S}(U-\omega_{H}U_{L}^{S})-T_{e}C_{T}U_{L}^{S}+\gamma_{L}C_{T}U_{H}^{S}$ (1.19)

$\frac{dU_{H}^{S}}{dt}=r{}_{A}A_{H}^{S}-r_{U}U_{H}^{S}-C_{M}\Lambda_{M}U_{H}^{S}-\mu_{d}U_{H}^{S}+\beta_{L}I_{S}(\omega_{H}U_{L}^{S})-(\gamma_{H}+\gamma_{L})C_{T}U_{H}^{S}$ (1.20)

$\frac{dP_{L}^{S}}{dt}=r{}_{T}T_{L}^{S}-r_{P}P_{L}^{S}-\mu_{d}P_{L}^{S}+\beta_{L}I_{S}(P-\omega_{H}P_{L}^{S})-T_{e}C_{T}P_{L}^{S}+\gamma_{L}C_{T}P_{H}^{S}$ (1.21)

$\frac{dP_{H}^{S}}{dt}=r{}_{T}T_{H}^{S}-r_{P}P_{H}^{S}-\mu_{d}P_{H}^{S}+\beta_{L}I_{S}(\omega_{H}P_{L}^{S})-(\gamma_{H}+\gamma_{L})C_{T}P_{H}^{S}$. (1.22)

The force of infection of schistosomiasis on the snail population, $\lambda_{S}^{V}$, was given by $\lambda_{S}^{V}=\beta_{s}((I_{L}^{S}+A_{L}^{S}+U_{L}^{S}+P_{L}^{S}+D_{L}^{S}+T_{L}^{S})+\varepsilon_{H}(I_{H}^{S}+A_{H}^{S}+U_{H}^{S}+P_{H}^{S}+D_{H}^{S}+T_{H}^{S}))$, where $\beta_{s}$is the schistosomiasis transmission rate from humans with low worm burdens to snails, $\varepsilon_{H}$is the schistosomiasis transmission rate from human with high worm burdens to snails relative to $\beta_{s}$. The probability that an infected mosquito survives the incubation period and becomes infectious is given by $\psi=e^{-\mu_{M}t_{incub}}$, where $\mu_{M}$ is the mosquito natural mortality rate and $t_{incub}$is the mosquito incubation period. The force of infection of malaria on the mosquito population, $\Lambda_{M}^{V}$, was given by $\Lambda_{M}^{V}=a(C_{D}(D+D_{L}^{S}+D_{H}^{S})+C_{A}(A+A_{L}^{S}+A_{H}^{S})+C_{U}(U+U_{L}^{S}+U_{H}^{S}))$, and the force of infection of malaria on the human population was given by$\Lambda_{M}=(amI_{M})b$, where $a$ represents the biting rate on humans by a female mosquito, $m$ is the density of mosquitoes per human, and $b$ is the probability of successful human inoculation upon an infectious bite. For the sake of simplicity, we assumed that the human population is constant over time with a natural death rate given by $\mu_{d}$ and a birth rate equal to

$\mu_{Pop}=\mu_{d}(S+I_{L}^{S}+A_{L}^{S}+U_{L}^{S}+P_{L}^{S}+D_{L}^{S}+T_{L}^{S}+I_{H}^{S}+A_{H}^{S}+U_{H}^{S}+P_{H}^{S}+D_{H}^{S}+T_{H}^{S})$. Effective treatment

(i.e., treatment which fully clears infection [4]) was assumed to be given to a proportion $f_{T}$of those who develop symptomatic disease.

We assumed that individuals entered the model susceptible and become infected with a low worm burden at a transmission rate$\beta_{L}$, and individuals with low worm burden may transition to high worm burden at a transmission rate $\beta_{H}=\omega_{H}\beta_{L}$. is the human high worm burden to snails transmission relative to $\beta_{L}$ . Likelihood of schistosomiasis transmission from humans to snails depends on worm burden. We assumed the individuals infected with low worm burden infect susceptible snails at a transmission rate$\beta_{S}$, and individuals with high worm burden infect snails at a transmission rate$\varepsilon_{H}\beta_{S}$. $\varepsilon_{H}$is the relative increase of the transmission rate to snails for high worm burden individuals relative to low worm burden individuals. Because rate of schistosomiasis reinfection is very high in endemic areas, we assumed that there is no natural recovery for *S. mansoni* infected individuals, and that without treatment infected individuals with a high worm burden will not transition to a low worm burden. We incorporated praziquantel treatment in the model by assuming that treatment has a$T_{e}$efficacy. We assumed that for individuals with high worm burden $T_{e}=\gamma_{H}+\gamma_{L}$where $\gamma_{H}$ is the cure rate and $\gamma_{L}$is the rate of becoming low worm burden upon treatment. A description of the model parameters is given in Table 1 in the main text and flow diagrams for the model are depicted in Figure S2.

### References:

1. Griffin JT, Hollingsworth TD, Okell LC, Churcher TS, White M, Hinsley W, Bousema T, Drakeley CJ, Ferguson NM, Basanez MG, Ghani AC: Reducing Plasmodium falciparum malaria transmission in Africa: a model-based evaluation of intervention strategies. PLoS Med 2010; **7**:e1000324.

2. Riley S, Carabin H, Marshall C, Olveda R, Willingham AL, McGarvey ST. Estimating and modeling the dynamics of the intensity of infection with *Schistosoma japonicum* in villagers of Leyte, Philippines. Part II: intentisity-specific transmission of *S. japonicum*. The schistosomiasis transmission and ecology project. Am J Trop Med Hyg 2005; **72**(6): 754-761.

3. Sokhna C, Le Hesran JY, Mbaye PA, Akiana J, Camara P, et al. Increase of malaria attacks among children presenting concomitant infection by Schistosoma mansoni in Senegal. Malar J 2004; **3**: 43.

4. Okell LC, Drakeley CJ, Ghani AC, Bousema T, Sutherland CJ. Reduction of transmission from malaria patients by artemisinin combination therapies: a pooled analysis of six randomized trials. Malar J 2008; **7**: 125.

# Supplementary figure


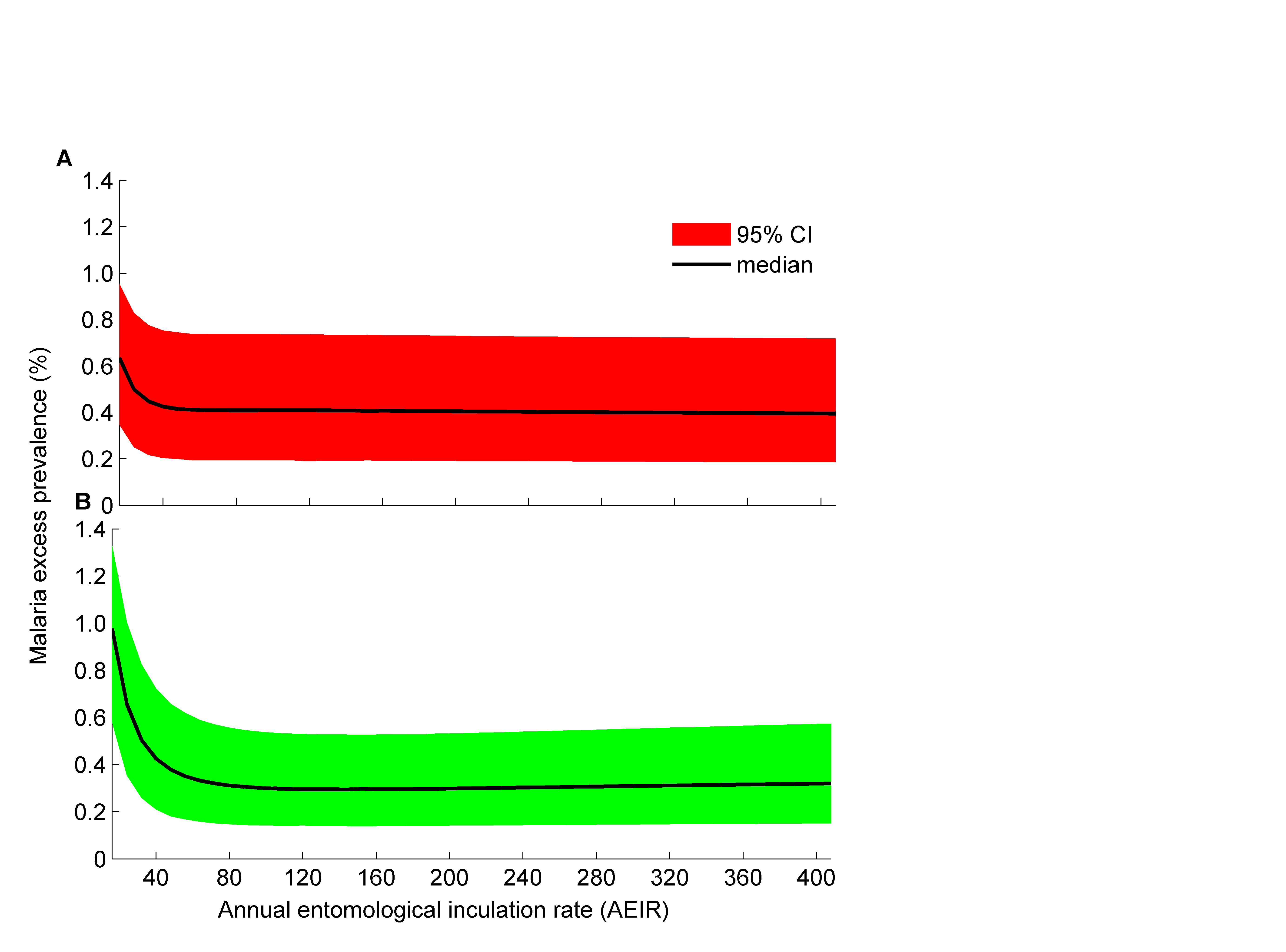


Figure S1: Difference in malaria prevalence attributable to *S. mansoni* at different level of malaria treatment coverage for a wide range of malaria and *Schistosoma mansoni* transmission settings. Malaria transmission settings were obtained by varying the AEIR, where as *S. mansoni* transmission settings were obtained by sampling schistosomiasis transmission parameters over the ranges of values given in Table 1. *S. mansoni* high worm burden is assumed to increase the risk of malaria infection by 85%$(C_{M}=1.85)$. Interaction between malaria and *S. mansoni* and the effect on (A) the difference in malaria prevalence attributable to *S. mansoni* for 70% malaria treatment coverage versus 50% treatment coverage, and (B) the difference between 90% malaria treatment coverage versus 70% coverage.
